# Mechanism of West Nile Virus Neuroinvasion: A Critical Appraisal

**DOI:** 10.3390/v6072796

**Published:** 2014-07-18

**Authors:** Willy W. Suen, Natalie A. Prow, Roy A. Hall, Helle Bielefeldt-Ohmann

**Affiliations:** 1 School of Veterinary Science, University of Queensland, Gatton, QLD, 4343, Australia; E-Mail: w.suen@uq.edu.au; 2 Australian Infectious Diseases Research Centre, University of Queensland, St. Lucia, QLD, 4072, Australia; E-Mails: n.prow@uq.edu.au (N.A.P.); roy.hall@uq.edu.au (R.A.H.); 3 School of Chemistry and Molecular Bioscience, University of Queensland, St Lucia, QLD, 4072, Australia

**Keywords:** West Nile virus, pathogenesis, neuroinvasion, blood-brain barrier, blood-CSF barrier, CSF-brain barrier, blood-spinal cord barrier, retrograde axonal transport

## Abstract

West Nile virus (WNV) is an important emerging neurotropic virus, responsible for increasingly severe encephalitis outbreaks in humans and horses worldwide. However, the mechanism by which the virus gains entry to the brain (neuroinvasion) remains poorly understood. Hypotheses of hematogenous and transneural entry have been proposed for WNV neuroinvasion, which revolve mainly around the concepts of blood-brain barrier (BBB) disruption and retrograde axonal transport, respectively. However, an over‑representation of *in vitro* studies without adequate *in vivo* validation continues to obscure our understanding of the mechanism(s). Furthermore, WNV infection in the current rodent models does not generate a similar viremia and character of CNS infection, as seen in the common target hosts, humans and horses. These differences ultimately question the applicability of rodent models for pathogenesis investigations. Finally, the role of several barriers against CNS insults, such as the blood-cerebrospinal fluid (CSF), the CSF-brain and the blood-spinal cord barriers, remain largely unexplored, highlighting the infancy of this field. In this review, a systematic and critical appraisal of the current evidence relevant to the possible mechanism(s) of WNV neuroinvasion is conducted.

## 1. Introduction

West Nile virus (WNV) continues to pose a significant disease burden in both human and animal populations, with new emerging or re-emerging strains appearing to be more neurotropic [1,2]. In 2012, the Center for Disease Control and Prevention (CDC) reported the highest number of human WNV cases in the U.S. since 2003 [3]. Of the 5674 cases, 51% were reported to be neuroinvasive, amounting to the highest number of human neuroinvasive cases caused by an arbovirus in U.S. history [2]. Prior to 2012, neuroinvasive cases per year accounted for less than 1% of symptomatic cases [4,5,6,7]. Meningitis, encephalitis and poliomyelitis appear to be the main pathological features in human disease [4]. Horses present with similar neurological disease, with neurological cases in WNV‑infected equids reported to be approximately 10% [6,8].

WNV is a mosquito-borne Flavivirus belonging in the family *Flaviviridae* [9]. The genome is a positive sense, single-stranded RNA which encodes a single polyprotein, that is post-translationally cleaved into three structural (C, prM/M, E) and seven non-structural proteins (NS1, NS2A, NS2B, NS3, NS4A, NS4B, NS5) [9]. A natural transmission cycle of WNV exists between mosquito vectors and reservoir hosts, such as aquatic birds [10]. Infection in incidental hosts, such as humans and horses, usually results in low level viremia and plays little role in the transmission cycle [11]. However, neurological disease can manifest in these hosts [12,13].

For a neurotropic virus, such as WNV, to invade the central nervous system (CNS), it must overcome both the extraneural and neural barriers present. This process is commonly referred to as neuroinvasion. Although extensive studies have been conducted on the virological and immunological mechanism of WNV neuroinvasion, the hypotheses that arose from these studies often require further validation. This review primarily aims to systematically appraise the current hypotheses of WNV neuroinvasion, notably the: (1) hematogenous and (2) transneural hypothesis of neuroinvasion. Largely unexplored and potentially promising areas of research, such as the blood-cerebrospinal fluid (CSF) barrier, the CSF-brain barrier and the blood-spinal cord barrier (BSCB), will also be evaluated in detail. Lastly, an overview of the important molecular determinants relevant to WNV neuroinvasion and the validity of the current rodent models will be addressed.

## 2. Hematogenous Route of Neuroinvasion

As viremia from peripheral infection with WNV is a common feature in various animal models (reviewed in [14]), the hypothesis of hematogenous dissemination of the virus into the CNS has been a common focus of investigation. According to current literature, viremia develops following peripheral replication locally in the dermis at the site of virus inoculation [15,16,17] and/or in the draining lymph nodes [15,18,19], both resulting in systemic dissemination of the virus. Viral presence in the CNS is commonly observed shortly thereafter, especially in studies using immunocompromised rodents [19,20,21,22,23,24,25,26,27,28,29]. This temporal kinetics of WNV infection has led many investigators to suspect the blood-brain interface, the blood-brain barrier (BBB), as the most likely route of neuroinvasion. For this reason, the role of the BBB in WNV neuropathogenesis is a common focus of investigation.

### 2.1. Blood-Brain Barrier

The BBB functions as a physical and immunological “shield” to prevent hematogenous pathogens and noxious substance from entering the brain, via its four main cellular components: endothelial cells and their basement membrane, astrocytes and their foot processes, microglial cells and pericytes [30,31]. The endothelium is the first line of defense against viral neuroinvasion. As such, *in vitro* endothelial models have been developed to study the mechanism of WNV translocation across this barrier (e.g., [32,33]). Mechanisms suggested for WNV include: (a) transcellular transport of virions across the infected endothelial cells (see Section 2.2) and (b) an increased permeability of the BBB, which can then facilitate a paracellular entry of the virus into the CNS parenchyma (see Section 2.4).

### 2.2. Transendothelial Viral Entry

Being a largely impermeable barrier under normal conditions, the BBB possesses many specialized transcellular transport systems that facilitate supply of essential nutrients into the brain [31]. But some of these strategies present an opportunity for neurotropic viruses to invade the CNS. *In vitro* experiments, using Japanese encephalitis virus (JEV), a close relative to WNV, demonstrated that transcytosis across both cerebral endothelial cells and pericytes via endocytic vesicles was achievable [34]. Specifically regarding WNV, Hasebe *et al.* [33] showed that virus-like particles (VLPs) could be transported from the apex to the basolateral sides of endothelial cells in culture, further supporting this transcellular transport hypothesis. The delay in the emergence of West Nile virions on the abluminal side from infected endothelial cell monolayer was also suggestive of a transcellular passage of the virus, instead of the more rapid process of paracellular diffusion [32]. 

**Figure 1:**
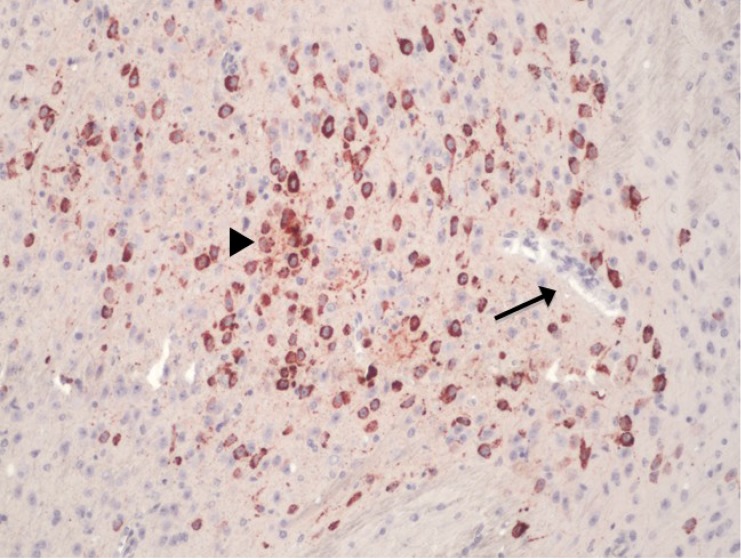
Immunohistochemical labelling with anti-flavivirus NS1 monoclonal on brain section from a Swiss CD1 mouse, infected with an Australian West Nile virus (WNV) subtype, WNV_KUN_. Despite ample neuronal infection (red stain, arrowhead), the endothelial cells are not infected (arrow). (Antibody binding was visualized using AEC substrate and the section counterstained with Mayer’s hematoxylin; magnification at 400×).

However, as observed by Hasebe *et al.* [33] and Dropulic and Master [35], the ability of virus to infect endothelial cells could be virus species specific and could even be strain specific. Furthermore, *in vivo* evidence of endothelial cell infection is lacking. One human case report noted the presence of JEV antigen in the endothelial cells of infected brains by immunohistochemical labeling [36]. However, on closer examination of the referred microphotomicrograph, the stained focus did not appear to be located in the endothelial cells, but was adjacent to it [36]. Indeed, brain endothelial cell infection by WNV can be absent even in the face of pronounced neuronal infection in mice (Figure 1). More importantly, brain endothelial cell infection is not a feature of WNV infection in the common target host, the equids [37]. This illustrates that the behaviour of *in vitro* grown endothelial cells can be quite different to that seen *in vivo*, especially when endothelial cells were cultured alone, as was the case in the studies by Verma *et al.* [32] and Hasebe *et al.* [33]. Therefore, the transcellular passage hypothesis suggested for WNV neuroinvasion requires further validation in an appropriate *in vivo* model.

### 2.3. Molecules for Blood-Brain Barrier Integrity

While *in vitro* infection of endothelial cells may result in transcytosis of virions, another hypothesis of neuroinvasion involves a paracellular migration of virions across a disrupted BBB. The mechanism by which this disruption occurs has been investigated again mostly in *in vitro* models, especially at the level of the endothelial junctions. The BBB possesses specialized tight junctions between brain endothelial cells. These junctions are made up of various junction proteins, mainly grouped as either tight junction proteins or adherens junction proteins. Claudins and occludins are the two major tight junction proteins responsible for the intercellular contact [30].

In general, most junctional proteins appear to have either an increased or stable genetic expression, but are present at a reduced level post-WNV infection both *in vivo* and *in vitro* (Table 1). This may suggest degradation of these proteins. The breakdown of these molecules have been suggested to be associated with BBB disruption, especially in the presence of pro-inflammatory cytokines, such as tumour necrosis factor (TNF)-α, interleukin (IL)-6, macrophage migration inhitory factors, and proteases, such as matrix metalloproteinases [38,39,40,41,42]. But in this area of WNV research, there is a lack of consistency in the *in vitro* methodology employed, such as the use of different cell lines, ranging from epithelial cells to endothelial cells from different anatomical origins, for infection [32,43,44]. This inconsistency is likely the reason for some of the discrepancies observed and listed in Table 1, such as the level of claudin-1 mRNA and protein levels. It is also interesting to note that the genetic expression of some of these proteins also differ between *in vitro* and *in vivo* studies (Table 1). This further highlights the difference in behaviour of cells grown *in vitro* as compared to those *in vivo*. 

**Table 1:** Genetic expression and protein levels of tight junction and adherens junction proteins post-WNV challenge in *in vitro* BBB models (endothelial cell) and *in vivo* models (brain).

Junction Proteins	mRNA	Protein Level
**Claudin-1**	↑ [32,44]; 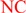 [42]	↑ [32]; 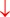 [41,42,43,44]
**Claudin-3**	↑ [44]	No information
**Claudin-4**	↑ [44]	No information
**Claudin-5**	NC [32]	No information
**ZO-1**	Mild ↑ [32,44]; 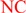 [42]	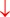 [41,42]; NC [43,44]
**Occludin**	Mild ↑ [32]; 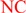 [42,44]	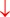 [42]; NC [44]
**JAM-A (JAM-1)**	↑ [44] 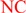 [42]	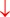 [42,44]
**Beta-catenin**	No information	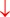 [42]
**Vascular Endothelial Cadherin**	No information	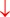 [42]

ZO-1: Zona occludens-1; JAM-A(JAM-1): Junctional adhesion molecule-A or 1; ↑ increased level; ↓ decreased level; NC no change; Y yes; N no; Words and symbols in red (e.g., 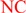 and 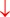) indicate findings supported by *in vivo* evidence [42].

In addition to tight junction molecules, cell adhesion molecules are another group of endothelial molecules of particular interests in BBB pathophysiology. These molecules may be upregulated in the presence of certain cytokines during inflammation (Table 2) [32,45,46]. Their functions include regulating leukocyte signalling and controlling movements of leukocytes in tissue during inflammation [47]. In the case of *in vitro* WNV infection of endothelium, intercellular adhesion molecule (ICAM)-1, vascular cell adhesion molecule (VCAM)-1 and E-selectin appeared to be upregulated (Table 2) [32,45]. The importance of ICAM-1 and E-selectin for leukocytic neuro-infiltration post-WNV inoculation is further supported by *in vivo* evidence [46,48]. Expression of certain integrins, such as very late antigen (VLA)-4 on LyC6^hi^ monocyte-derived macrophages, may also be important in aiding migration of particular leukocytes into the CNS *in vivo* [49]. While some of these studies have suggested that cell adhesion molecules may play a role in facilitating migration of peripherally infected leukocytes into the CNS [32,46], no direct evidence is available to demonstrate this phenomenon (see Section 2.4.2 for the “Trojan Horse” route of neuroinvasion).

**Table 2:** Genetic expression and protein levels of cell adhesion molecules in endothelial cell (*in vitro*) and brain (*in vivo*) post-WNV infection.

Cell adhesion molecules	mRNA	Protein levels
**ICAM-1**	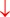 [48]	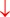 [45,48]
**ICAM-3**	NC [32]	No information
**VCAM-1**	↑ [32]; 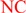 [48]	↑ [32,45]; 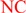 [48]
**E-Selectin**	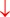 [32,48]	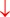 [45,48]
**P-Selectin**	No information	NC [45]
**PECAM**	NC [32]	No information

↑ increased level; ↓ decreased level; NC no change; Words and symbols in red (e.g., 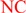 and 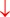) indicate findings supported by *in vivo* evidence [48].

### 2.4. Paracellular Viral Entry

As a consequence to BBB leakage, plasma protein and inflammatory cells could migrate across paracellular junctions between endothelial cells into the brain parenchyma. Two methods of viral entry, as a result of BBB breakdown, have therefore been hypothesized: (1) via passive diffusion as cell-free virions, or (2) via infected-leukocytic trafficking, *i.e.*, the “Trojan Horse” method.

#### 2.4.1. Via Passive Diffusion

The hypothesis of passive diffusion of cell-free virions across a more permeable BBB has been put forth in several reviews [1,50,51]. However, searches in two separate literature databases (Pubmed and Web of Science) for this hypothesis failed to yield any study that directly demonstrated actual diffusion of WNV across the BBB.

Referring to the reviews by Clark *et al.* [1], Sips *et al.* [51] and Samuel and Diamond [50], the studies that were cited in support of this particular hypothesis for WNV, did not actually demonstrate this diffusion phenomenon. For example, Sips *et al.* [51] cited Verma *et al.* [32]. However, this latter study actually demonstrated the contrary. The time taken for WNV to cross the more permeable BBB fitted better with the idea of a transendothelial transport of the virus, rather than via passive diffusion [32]. This was further validated by the lack of FITC-dextran diffusion across the endothelial monolayer post-WNV infection [32]. Similarly, Samuel and Diamond [50] cited Kramer-Hammerle *et al.* [52], which was a review on the mechanism of neuroinvasion by HIV-1. As well as referring to a virus in a different family, the relevant section in the paper by Kramer-Hammerle *et al.* on paracellular diffusion across the BBB lacked any referencing [52]. Clark *et al.* [1] cited reference [39]. While this study investigated the role of TLR-3 and its downstream cytokines in WNV neuropathogenesis, it did not directly demonstrate cell-free virion migration across BBB via the paracelluar junction. It was only hypothesized [39].

While the notion of passive diffusion across a permeabilized BBB is conceptually possible, in light of the lack of evidence, this hypothesis of neuroinvasion requires further investigation. Until solid evidence is produced to show serum-derived cell-free virions diffusing across the endothelial junctions of the BBB, this hypothesis should be mentioned with caution. Also while many *in vivo* studies concluded that there is a correlation between BBB disruption and WNV infection of the brain, it is unclear the direction of this potential causal relationship. Studies such as that by Roe *et al.* [42] showed that the timing of increased BBB permeability does not precede viral neuroinvasion. Indeed, WNV infected neurons are capable of producing pro-inflammatory cytokines, such as IL1β, IL-6, IL-8 and TNF-α, *in vitro*, which may disrupt the BBB [53]*.* So perhaps the post-infection permeability changes in the BBB could just be a result of an initial viral neuroinvasion via one of the other routes, such as the transneural option (see Section 3)? This ultimately questions the true relevance of the BBB permeability in the process of WNV neuroinvasion, especially if it may only be an effect rather than a cause of viral entry into the CNS.

#### 2.4.2. Via a “Trojan Horse” Mode of Entry

The “Trojan Horse” hypothesis, as proposed in many reviews [51,54,55] and primary studies [32,41,56,57], involves the trafficking of infected leukocytes across a permeabilized BBB, via the paracellular junction, into the CNS parenchyma. Garcia-Tapia *et al.* [56] suggested that WNV infected Langerhan cells migrated from the site of inoculation to draining lymph node, where infection could then be relayed to mononuclear cells, such as monocytes and certain subsets of CD4^+^ lymphocytes. As hypothesized from a later study, Garcia-Tapia *et al.* suggested that the expression of lymphocyte and monocyte chemoattractants, such as IP-10 (CXCL10) and MCP-5 (CCL12), respectively, in WNV infected brains, post-footpad inoculation, could recruit peripheral mononuclear cells into the perivascular space in the CNS [16]. Here, the recruited leukocytes could produce pro-inflammatory cytokines, such as TNF-α and interleukins, which as mentioned above could compromise the BBB integrity [16,39]. Infected monocytes/macrophages and CD4^+^ lymphocytes could also facilitate productive viral replication in this region [56,58], providing a source of infection for brain microvascular endothelial cells, which in turn may exacerbate the BBB permeabilization via the degradation of inter-endothelial tight junctions and upregulation of CAM expression [32,46]. Further recruitment, margination and transmigration of infected leukocytes across the paracellular junction of the BBB could result in viral neuroinvasion and dissemination [16]. 

While studies have demonstrated co-localization of WNV antigen in CNS-infiltrating leukocytes [46,59], there is no current evidence to show that peripherally infected leukocytes have the ability to traverse into the CNS. Wang *et al.* have shown that WNV antigen can be found in splenic and CNS‑infiltrating CD4^+^ and CD8^+^ T cells [59]. However, the peripheral lymphocytes were isolated at a time when viral neuroinvasion had already occurred (day six post-infection) [59]. Thus, it is unclear whether the peripheral leukocytes were infected prior to entering the CNS. Indeed, it is open to interpretation whether the observed WNV antigen-positive leukocytes in the CNS were a consequence of “Trojan Horse” viral neuroinvasion or post-phagocytosis of WNV-infected neurons. Furthermore, the function and trafficking behaviour of WNV infected leukocytes have not been examined, as noted by King *et al.* [60] and Bielefeldt-Ohmann *et al.* [61]. Therefore, further testing of this hypothesis is required. Real-time fluorescent imaging techniques involving fluorescent reporters, such as mCherry (e.g., [62]) or dsRed (e.g., [63]) in WNV infectious clones may provide new tools for demonstrating the behaviour of these peripherally infected leukocytes *in vitro* and *in vivo*.

## 3. Transneural Route of Neuroinvasion

Transneural invasion of the CNS by WNV is an emerging area of investigation. As compared to the literature available on the hematogenous mechanism of neuroinvasion, the number of studies that investigated the hypothesized transneural mechanism is under-represented. Overall, two neuroanatomical areas have been hypothesized to be involved in this mechanism: (1) from the peripheral somatic nerves into the CNS, or (2) from the olfactory nerves into the CNS.

### 3.1. From Peripheral Nervous System (Somatic Nerves) to Central Nervous System

Through direct injection of WNV inoculum into the sciatic nerve, investigators have shown that transneural spread of the virus from the peripheral nervous system (PNS) to the CNS could be a putative route for neuroinvasion [64,65]. Both studies utilized a direct intrasciatic nerve inoculation model in hamsters to demonstrate viral antigen in the spinal cord post-inoculation. Wang *et al.* [65] specifically noted that WNV appeared to travel preferentially up motor nerves rather than sensory by their observation that WNV antigen was present in only the motor neurons, rather than the dorsal root ganglia. *In vitro* experiments showed that intact axons were required for intra-neuronal spread and transmission of virions between neurons appeared to be via transfer across synapses [64]. Compartmentalized neuron methods also showed that bidirectional spread of WNV was possible [64]. Samuel *et al.* [64] speculated that the anterograde axonal transport would play a role in disseminating the virus across the nervous system. 

However, neither study investigated brain viral load or pathology following intrasciatic nerve inoculation [64,65]. This potentially questions the ability of this model to demonstrate the complete transneural transport of WNV to the brain. Moreover, the sciatic nerve contains a mixture of sensory and motor fibres [66]. Delineation between the sensory and motor pathway was only achieved by indirect means in the study by Wang *et al.* [65]. For conclusive evidence that WNV does indeed preferentially escalate up the motor nerve fibres, double immunolabelling of sensory/motor nerves and WNV antigen with specific anti-sensory or motor nerve antibodies, such as anti-CV2 [67] for sensory nerve fibres, at an appropriate time-point post-infection is needed. Moreover, WNV infection of the autonomic nervous system in hamsters further highlights a potentially broader tropism than just motor neurons [68].

This area of research is under-represented and as such, there remain several gaps in this hypothesized route of WNV neuroinvasion. Applying approaches used in studies of other well‑characterized neurotropic viruses, such as rabies virus and herpes simplex virus, molecular investigation into the involvement of the intracellular dynein and kinesin motor system in retrograde axonal transport will help advance the understanding of this hypothesized route. Brault *et al.* has already noted an interaction between the ectodomain of the WNV membrane protein (ectoM) and Tctex-1, a light chain on the human dynein protein, during late stage viral replication [69]. While the neuroinvasive aspect of this ectoM-Tctex-1 interaction was not the focus of the study, the interaction appeared to be important for internal transport of viral particles within infected cells [69]. The relevance of ectoM for WNV in retrograde axonal transport should therefore be investigated further *in vivo*, especially when suggestive observations of this route of neuroinvasion are emerging from WNV foot-pad challenged studies [15,57,70].

### 3.2. From Olfactory Nerve to Olfactory Bulb

As evident in a few *in vivo* challenge studies using neurotropic flaviviruses inoculated by either an intraperitoneal [71], subcutaneous (footpad) [15] or intranasal [72] route, the initial detection of viral antigen in the brain began in the olfactory bulb. As suggested from the sequential distribution of viral antigen along the olfactory pathway, a general hypothesis of the olfactory route of neuroinvasion has been proposed for Flaviviruses.

In some studies using footpad inoculation, viremia preceded the initial appearance of viral antigen in the olfactory bulb [15,73]. This suggested a potential hematogenous source of infection, likely initiated at the level of the olfactory neuroepithelium, which contains fenestrated blood vessels [74]. But the point of entry at this level is still controversial. Monath *et al.* [71], in a study that is commonly cited in WNV literature, suggested that St. Louis encephalitis virus (SLEV) traversed from the dendrites of the bipolar olfactory neurons in the olfactory neuroepithelium. However, detection of viral antigen in this area was not common [75]. When viral antigen was present, the results were often biased, due to the use of an intranasal route of inoculation [72]. Inconsistent sampling of the olfactory neuroepithlium in challenge studies was another source of bias.

While the initial site of virus entry remains elusive, the rostral-caudal spread of flaviviral antigen, and specifically for WNV, from the olfactory bulb to the remaining structures of the brain has been documented in a few studies [15,72,73]. This was observed by mapping the sequential distribution of viral antigen in brains of infected animals terminated at various time points [15,72,73]. Since trans- and inter-neuronal spread was possible across synapses, as demonstrated *in vitro* with WNV [64], viral dissemination along the brain neural circuits after olfactory bulb invasion may explain the temporal pattern of virus distribution, as suggested by McMinn *et al.* for Murray Valley encephalitis virus (MVEV) [73].

However, on analysing the data in a selection of these studies, the increased spread of viral antigen across the brain occasionally coincided with a concurrent persistent viremia (e.g., [73]). This viremia may influence the virus distribution in the brain in these later stages of the infection course. Indeed, hematogenous viral neuroinvasion via a permeabilized BBB may have occurred after an initial invasion via the olfactory route, as suggested in Section 2.4.1. This presents an alternative explanation to the inter-neuronal spread hypothesis for the apparent rostral-caudal “spread” of infection over time in the brain. To further support this, when concurrent viremia was not persistent or negligible, as in the case of the attenuated BHv1 strain of MVEV [73], the spread of the viral infection was halted and confined to just the olfactory lobe. Again, real time fluorescence imaging techniques may provide a novel tool for investigating the movement of tagged viral particles post-invasion into the olfactory bulb and elsewhere *in vivo.*

Lastly, report of WNV antigen in the olfactory lobes in horses and humans is rare. Viral antigen was primarily observed in mid to hindbrain and spinal cord in horses and humans [37,76]. However, post-mortem examination was often conducted on cases that were already showing symptoms, at which time initial viral neuroinvasion into the olfactory bulb may not have been captured. Inconsistent sampling of the olfactory bulb in these hosts may also lead to under-reporting of viral antigen in this region. Therefore the relevance of this route of neuroinvasion in incidental hosts remains unknown.

## 4. Multiple Routes of Neuroinvasion

Drawing together the conclusions from the above *in vivo* and *in vitro* studies, the route of WNV neuroinvasion may be much more complex than one distinct path from the peripheral site of inoculation to the CNS. Hence the hypothesis of multiple routes of neuroinvasion has been proposed [15,42,57]. In these studies, suggestive signs of utilization of more than one route were observed. Brown *et al.* [15] noticed the sites of initial WNV neuroinvasion occurred in both the olfactory bulbs and spinal cord, following footpad injection in mice. This might imply transneural CNS invasion via both the peripheral somatic nerve, ending in the spinal cord, and the olfactory nerve, ending in the olfactory bulb. Similarly, Hunsperger *et al.* [57,70] hypothesized that WNV utilized axonal retrograde transport to gain entry into the spinal cord and brainstem early in the course of the infection. But they also hypothesized the involvement of the blood-CSF barrier in the choroid plexus in neuroinvasion, though the validity of this particular point of the study can be challenged (see Section 5) [57,70]. Finally, Roe *et al.* observed a biphasic pattern of WNV CNS infection [42]. They detected an initial viral entry without compromising the BBB, followed by a second wave of CNS re-infection, possibly achieved by the “Trojan Horse” mode of neuroinvasion [42]. While the studies mentioned above concluded with different interpretations, they have nevertheless observed some signs indicative of multiple points of WNV entry into the CNS. Indeed, the traditional question of which singular route is responsible for viral neuroinvasion should now be reconsidered and expanded to which multiple routes are involved.

## 5. New Avenues for Research

While the focus of many WNV pathogenesis studies has been directed mainly at the BBB and the transneural transport theories, there remain several routes of viral entry into the CNS that have received little attention. In the brain, the role of the blood-CSF and the CSF-brain barriers in WNV neuroinvasion is unknown. In fact, the “BBB” have occasionally been inaccurately used to describe the blood-CSF barrier [77] and the arachnoid (meningeal) barrier [42]. This indicates a lack of consensus in the interpretation of terms “blood-brain barrier”. A distinction of the different barrier mechanisms in the CNS is important, since there are some significant differences that will ultimately impact on the success or failure of viral neuroinvasion (Table 3).

The main distinction between the BBB proper and the other barrier mechanisms is the presence of the support cells surrounding the former, suggesting a functionally more complex barrier in the blood‑brain interface in the cerebrum. The difference in the components of the barriers may also imply a difference in the susceptibility of breakdown. In fact, the arachnoid (meningeal) barrier has been shown to be the first barrier to be disrupted four hours post-intracisternal injection of IL-1β and LPS in rats [78]. The blood-CSF barrier of the choroid plexus and the BBB were not affected within the time frame of the study [78]. The significance of the arachnoid (meningeal) barrier in relation to viral neuroinvasion has not been explored.

**Table 3:** Characteristics of the different barrier mechanisms in the brain.

Barriers	Blood-brain (neurovascular unit)	Blood-CSF (ventricular system)	Blood-CSF (arachnoid/meningeal)	Inner CSF-brain	Outer CSF-brain
**Anatomical location**	Cerebral blood vessels	Choroid plexus and CVO blood vessels	Dural +/− pial blood vessels	Ependyma of the ventricular system	Pia mater
**Cellular components**	Endothelial cells (non-fenestrated) Pericytes Astrocytes Microglia Neurons	Endothelial cells (fenestrated) Epithelial cells of choroid plexus Tanycyte-like cells of CVO	Endothelial cells (fenestrated dural vessels) Arachnoid epithelial cells	Neuro-ependymal cells (the ventricular system)	Glial foot processes Pial epithelial cells
**Restriction point**	Endothelial cells (tight junction)	Epithelial and tanycyte-like cells (tight junction)	Arachnoid epithelial cells and pial blood vessel endothelial cells (tight junction)	Neuro-ependymal cells (strap junction, only in immature brains)	Glial foot processes (various junctions, only in immature brains)

CVO, circumventricular organs. This table is a synthesis of several reviews [79,80,81,82,83,84].

**Figure 2:**
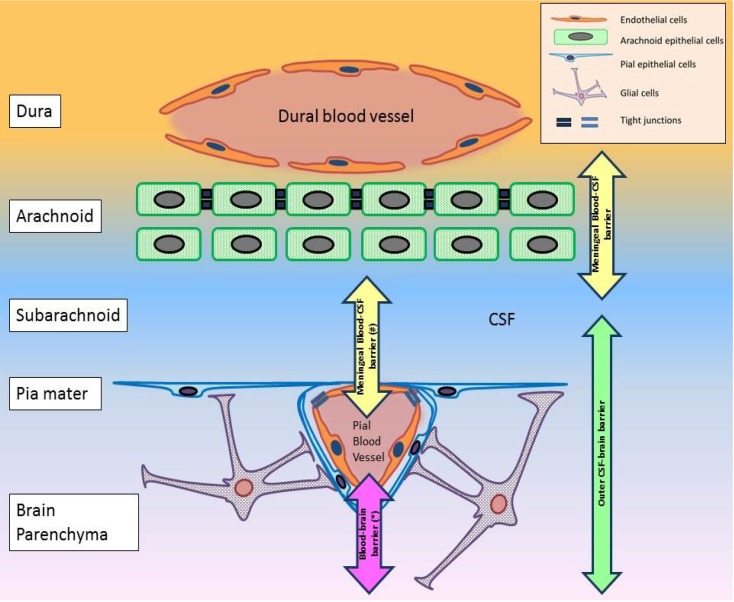
The different barrier mechanisms in the meninges. The meninges consists of the dura, arachnoid, subarachnoid and the pia mater. (*) Pial blood vessels differ from cerebral blood vessels in two regards. The former lacks astrocytic ensheathment that is observed in the latter [85]. Tight junctions between pial endothelial cells are also different [85]. So the “blood-brain barrier” in this region differs from the BBB proper in the cerebrum. (#) A blood-CSF interface possibly exists between pial blood vessels and CSF in the subarachnoid. But no information can be found on this hypothetical interface. This diagram has been constructed based on information collected from [78,80,81,82,83,86].

However, the meninges is a complex region. The following interfaces are present in the meninges:

1. A blood-CSF barrier at the level of the outer arachnoid layer (Figure 2) (e.g., [78,83]).
2. A blood-brain interface in the pial microvessels (Figure 2) [81].
3. A hypothetical blood-CSF interface in the pial blood vessels (Figure 2).
4. An outer CSF-brain interface in the pia mater (Figure 2) [80].

Of the above interfaces, only the first and occasionally the second were referred to as the arachnoid (meningeal) barrier, even though pial microvessels are not situated in the arachnoid (Figure 2). The pia mater, with the glial foot processes underneath, forms the outer CSF-brain barrier, which is only impermeable in the immature brain [80]. There is no report of a blood-CSF interface at the level of the pial blood vessel. But it is a possible interface given its proximity to the subarachnoid CSF (Figure 2). Such complex architecture of the meninges creates challenges in interpreting findings from studies involving this region, such as that by Ichikawa *et al.* [78]. Precise attention to the separate interface should be attempted, as the significance of each interface will be different given the different cell types involved. But despite this, the meninges is a convergence point of several interfaces. As such, a unique interaction may be present between them. Whether this interaction generates an environment conducive for viral neuroinvasion remains to be determined.

While little is known about the involvement of the arachnoid (meningeal) barrier in viral pathogenesis, the blood-CSF barrier in the choroid plexus has received some attention in simian immunodeficiency virus (SIV) [87] and highly pathogenic avian influenza virus (H7N1) research [88]. Both studies have highlighted virus entry via this route is possible, either by a “Trojan horse” method [87] or a direct involvement with cell surface receptors of the choroid plexus epithelial cells [88]. For WNV, one research group has suggested the involvement of this barrier [57,70]. But their observation of WNV antigen in the nuclei of choroid plexus epithelial cells should be interpreted with caution, especially when immunohistochemistry was performed using a polyclonal primary antibody [57]. One cannot rule out the possibility of non-specific staining artifact in this case. Nevertheless, the choroid plexus contains fenestrated blood vessels with restriction points only at the level of the choroid plexus epithelium, and therefore is a theoretically possible route for viral neuroinvasion [79]. A recent investigation of the circumventricular organs has led to the detection of a blood-CSF barrier in association with the fenestrated blood vessels in these regions [84]. The barrier consists of a specialized type of ependymal cells, named tanycyte-like cells, held together by tight junctions [84]. Once perceived as a free exchanging area of CSF and blood, this barrier in CVOs may now open a new avenue for viral neuropathogenesis investigation.

In the literature, the CSF-brain interface has been broadly split into two barriers: inner and outer CSF-brain barrier (Table 3). An age—related permeability difference in these barriers has been suggested by Saunders *et al.* [80]. This is an interesting note, since it suggests that depending on the age of the animal, viruses that have successfully passed into the CSF from the blood, for example SIV as mentioned above, may not necessarily enter the brain parenchyma [80]. Implications of this dynamic behaviour of the CSF-brain barrier on viral neuroinvasion have not been examined, even though an age-related difference in susceptibility has been observed post-WNV infection in mice (e.g., [89,90]). Furthermore, the pial membrane, which is part of the CSF-brain interface, is also not a completely uninterrupted membrane lining the outer surface of the neural axis [86]. Fenestrations have been detected by scanning electron micrograph at the *conus medullaris* and the nerve roots in the spinal cord [86]. These findings present another set of vulnerable points of entry for neurotropic viruses, such as WNV.

In fact, apart from investigations into the transneural hypothesis of neuroinvasion, the spinal cord has been a largely neglected topic in WNV neuropathogenesis research. But there are some important distinctions in this region as compared to the brain. The blood-spinal cord barrier (BSCB) is a good example. While the BSCB shares the same cellular components as the BBB, emerging evidence indicates that it is more permeable than the BBB proper, potentially due to the lower number of pericytes [91] and/or the expression of different junctional proteins between endothelial cells [92] in the BSCB. Cytokines, such as IFNs and TNF-α, which can be neurotoxic (e.g., [53,93]), have also been reported to diffuse across the BSCB more readily than the BBB [92]. This potentially implies that neuropathology in the spinal cord can be instigated without the need for viral neuroinvasion, if neurotoxic cytokines can diffuse into the neuropil from a hematogenous source, such as during an episode of cytokinemia. Indeed, this example of the BSCB emphasizes the need for a better delineation of the role of the spinal cord in the neuropathogenesis of WNV.

Overall, there are some major gaps in the current knowledge of how WNV enters the CNS. As indicated in this section, it may be time to consider switching the current research focus away from the popular areas, such as the BBB, and towards the unexplored avenues.

## 6. Molecular Determinants for WNV Neuroinvasion

Historically, WNV mainly manifested as a febrile disease, termed West Nile fever, in humans [94]. Since the mid-1990’s, larger WNV outbreaks with more severe neuroinvasive cases have been reported in humans globally [8]. This also coincided with an increase in morbidity and mortality in equine and avian species [8,94]. Several studies have characterized the evolution of the WNV genotype, particularly strains in lineage 1 and 2 [95,96]. A consistent shift in both lineage 1 and 2 WNV strains to a more neuroinvasive phenotype appeared to have taken place [96]. Extensive efforts into locating virulence determinants in lineage 1 strains are summarized in Table 4. For lineage 2 strains, amino acid positions 159 for E, 338 for NS1, 126 for NS2A, 421 for NS3, 20 for NS4B and 254 for NS5 appeared to be important regions that differentiated attenuated and virulent strains [96].

While virus genotypes that produced high mortality and/or high CNS viral burden after peripheral inoculation have been identified as highly neuroinvasive (e.g., [97]), several important points should be considered when interpreting these findings. The observation by Appler *et al.*, that infectious WNV can be isolated from brains of surviving mice one month post-infection, highlights some limitations associated with using lethality studies and CNS viral burden to identify virulence determinants [98]. It indicates that the process of neuroinvasion does not always result in a high CNS viral load and high mortality. As suggested by Morrey *et al.*, death in rodents is highly correlated with respiratory insufficiency likely attributable to infection/lesion in the respiratory center of the CNS [99]. So this suggests that the distribution of CNS infection/lesions may be a more important determinant of mortality, and hence virulence, than the overall CNS viral burden. This stresses the need for complete work-up for all survival studies involved in identifying virulence determinants. 

Likewise, host factors that affect WNV dissemination or productive infection in the CNS will ultimately affect the viral burden. So the term “neuroinvasiveness” should probably be used with caution, since an increased CNS viral load could be attributable to an increased efficiency in viral dissemination and/or productive infection of cells in the CNS, even when the neuroinvasive potential of the virus has not changed. Therefore, it is important for virulence studies to delineate the contributions of the above processes when high CNS viral load is observed post-peripheral inoculation. Currently, the magnitude of biases presented by the above factors on virulence studies has not been thoroughly assessed.

While the determinants listed in Table 4 may be relevant for the overall *in vivo* virulence phenotype, their precise role in the process of WNV neuroinvasion is not clear. Some efforts have been placed in characterizing the relevant viral factors specifically for this. Table 5 provides a summary of the current understanding in these potential determinants, particularly via their role in interacting with the host cell surface for cellular uptake, or intracellular proteins for intracellular trafficking, immune evasion, instigating neuroinflammation or breakdown of junctional proteins. However, as compared to other important neurotropic viruses, such as rabies virus, the characterization of the viral mechanism(s) for WNV neuroinvasion is only at its infancy.

**Table 4:** Summary of WNV lineage 1 *in vivo* virulence determinants.

Region	Position (amino acid/nucleotide)	Mutation studied	Effect on *in vivo* virulence	WNV strain	Citations
prM	141 ^#^	Isoleucine → Threonine	Attenuation (birds)	NY99 and TM171-03-pp1 (Mexico)	[100]
E	Whole prM-E	prM-E of ETH76a inserted into NY99 backbone	Attenuation (mice)	ETH76a and NY99	[97]
	154–156 (Glycosylation motif)	Asparagine → Serine (154) ^$^	Attenuation (mice)	NY99	[101]
		Serine → Proline (156) ^#^	Attenuation (birds)	NY99 and TM171-03-pp1 (Mexico)	[100]
NS1	250	Proline → Leucine	Attenuation (mice)	Kunjin	[102]
	130-2, 175 and 207 (Glycosylation sites) ^$^	Asparagine → Serine/Glutamine/Alanine (130-2); Asparagine → Alanine (175 and 207)	Attenuation (mice)	NY99 (382-99)	[101,103]
NS1' and NS2A	Frameshifting motif (NS2A)	Silent mutation	Partial attenuation (mice)	Kunjin	[104]
	30	Alanine → Proline	Attenuation (mice)	Kunjin	[104,105]
NS3	249 (Helicase domain)	Threonine → Proline	Enhanced virulence (birds)	KN-3829 (Kenyan) and NY99 (382-99)	[106]
NS4A (and 2K)	9 (2K protein)	Valine → Methionine	Enhanced virulence(mice and birds)	North American (Texas 2003 and NY 2002 and 1999)	[107,108,109]
NS4B	38, 166 and 480	Proline → Glycine (38); Threonine → Isoleucine (116); Asparagine → Histidine (480)	Attenuation (mice)	NY99	[110,111]
	249	Glutamate → Glycine at 249	Attenuation (mice and bird)	North American (Texas 2003 and NY 2002 and 1999)	[109,112,113]
5'UTR	50–52 ^@^	NY99’s 5'UTR substitutes KUN’s	Enhanced virulence (mice)	Kunjin and NY99	[90]

^#^ these two prM and E mutations work in conjunction; ^$^ these two E and NS1 mutations work in conjunction; ^@^ nucleotide positions; → amino acid substitution.

**Table 5:** Summary of determinants relevant for WNV neuroinvasion.

Region	Position (amino acid/nucleotide)	Mutation studied	Effect of mutation	*In vivo/vitro*	WNV strain	Citations
C	Unknown	N/A	Immune evasion	*In vitro*	NY99	[114]
	Unknown	N/A	Neuroinflammation	*In vivo* (rats)	NY99	[115]
	Unknown	N/A	Tight junction protein degradation	*In vitro*	NY99 (385-99)	[43]
prM/M	ectoM domain	N/A	Interacts with Tctex-1	*In vitro*	IS-98-ST1 and other flaviviruses	[69]
E	156–160 (αA’ structure of domain I)	N/A	Affinity to DC-SIGN/DC-SIGNR	*In vitro*	NY99 and Egypt	[33]
	RGD motif (domain III)	N/A	Interacts with cellular integrin	*In vitro*	Sarafend and DEN-2 (New Guinea)	[116]
NS2A	30	Alanine → Proline	Reduction in immune evasion via IFN-β and unknown antiviral pathway	*In vitro and in vivo (mice)*	Kunjin	[105]
NS3	365 (NTPase domain)	Serine → Glycine	Immune evasion via resistance to OAS1b	*In vitro*	NY99 (382-99)	[107]
NS4B	22 and 24	N/A	Immune evasion by inhibiting IFN cascade	*In vitro*	Subgenomic WNV replicons (without structural genes) derived from KUN	[117]
	38	Proline → Glycine	Attenuation in neuroinvasiveness (mice) due to enhanced IFN and T cell response	*In vivo*	NY99	[110,111]
NS5	653	Serine → Phenylalanine	Immune evasion by inhibiting JAK-STAT pathway	*In vitro*	Kunjin	[118]

→ amino acid substitution; N/A no mutation was investigated.

## 7. Animal Models for WNV Neuroinvasion

While the studies in Table 5 draw attention to important determinants, relevant for WNV neuroinvasion, a major drawback to these findings is the over representation of *in vitro* evidence and a general deficiency in *in vivo* validation of the suggested mechanism. When *in vivo* studies were performed, the model of choice was often rodents as it represents an economical and convenient model. However, in several aspects, the disease profile of WNV infection in rodents is not representative of that in incidental hosts (Table 6). 

**Table 6:** Comparison of the general disease profile between rodent hosts, such as mice and hamsters, and intermediate hosts, such as horses and humans.

Feature	Mouse	Hamster	Horse	Human
**Peak Viremia **	Moderate (~10^4^ PFU/mL) [19,42,57,70]	Moderate (10^5^ TCID_50_/mL) [119]	Low (10^1−3^ PFU/mL) [120]	Very Low (only detectable by real-time RT-PCR) [121,122]
**Peripheral tissue tropism**	Lymph nodes Spleen Kidneys [16,19,123]	Kidneys and urine Spleen and lungs (occasionally) [124,125,126]	Rare [127]	Testicular tissue Urine Kidney and urine Spleen [128,129,130]
**Distribution of CNS Lesions **	Widespread: - cerebral cortex - hippocampus - brainstem - spinal cord (e.g., [16])	Widespread: - cerebral cortex - basal ganglia - hippocampus - cerebellar cortex - brainstem [119]	Commonly: - hindbrain - spinal cord Rarely: - cerebral cortex - cerebellum [13,37]	Commonly: - mid-brain - brainstem - spinal cord Occasionally: - cerebral cortex - hippocampus - cerebellum [12,76,130,131]
**WNV infection in the brain **	High level (~10^5−7^ PFU/g) [16,19,57]	High level (IHC) [119]	Low level to absent (IHC) [13,37,132]	Low level unless immuno-compromized (IHC) [76]
**Mortality rate **	➢ 35 to 45% of infected (C57BL6) [19] ➢ 100% of infected (CD1 Swiss) [89]	➢ ~40% of infected [119,126]	➢ ~10 to 57% ^$^ of clinical cases (WNV strain dependent) [13,37,89]	➢ ~10% of neuroinvasive cases accounting for less than 1% of infected persons [133,134]

^$^ The mortality rate in the horse does not distinguish the number of cases euthanatized or died naturally.

The relevance and implications of these differences may be profound. One may contend that if the viremia is different between rodents and the incidental hosts, the route of viral entry into the CNS may differ, as different quantity of hematogenous virions will be delivered to the various CNS barriers. Another important distinction is the difference in the severity of CNS infection between these species. 

The majority of human cases that succumb to WNV infection exhibit only minimal to mild neuronal infection in the CNS, unless pre-existing co-morbidities coincide with WNV infection, in which case profound CNS infection and increased peripheral tropism can occur [12,76,135]. While this latter scenario may appear similar to that observed in the WNV infected rodents, as suggested by Diamond *et al.* [136], it may be inappropriate to utilize an immunocompetent rodent model to study the pathogenesis of WNV infection in immunocompromised humans. The behaviour of both branches of immunity, innate and adaptive, of an immunocompetent rodent is unlikely to represent that of an immunocompromised human. Furthermore, as highlighted by the genome-wide expression analysis study by Seok *et al.*, the gene expression profile of the murine immune response poorly correlates with that of humans during acute inflammatory stress [137]. An ideal pathogenesis model for the human WNV disease should therefore have a fairly resistant disease profile when immunocompetent and should only exhibit widespread CNS infection and peripheral tropism when the immune system is compromised by either co-morbidities or old age. 

The current “gold-standard” animal model for human diseases is non-human primates (NHP). After experimental infection with WNV, immunocompetent rhesus macaques and marmosets do not show clinical signs, nor can infectious virus or viral RNA be detected in the CNS [138,139]. Viremia in these species are also undetectable or transient and of low magnitude, 10^2−3^ TCID_50_/mL [138,139]. While studies into the effects of WNV infection in immunosuppressed NHP have not yet been conducted, the disease course seen in NHP is more likely to reflect that in humans than that seen in the rodent models. However, since the NHP model is associated with substantial costs, ethical concerns and logistic difficulties, an alternative small animal model should be explored. Currently, efforts have been placed in establishing a New Zealand white rabbit challenge model for a novel Australian WNV subtype [140]. Other possible alternatives for investigation include guinea pigs and ferrets, which have been used as small animal models for immunologic investigation with JEV [141] and other infectious agents such as influenza [142].

Similar to the human disease, horses that succumb to natural or experimental WNV infection also showed scant neuronal infection in the CNS [13,37]. So the role of immunopathology in contributing to the associated encephalomyelitis has been suggested [13]. Whether co-morbidities play a role in contributing to equine mortality is an area requiring further investigation. However, caution should be exercised when interpreting mortality in horses, since death due to euthanasia *versus* natural death is rarely distinguished in the literature. The effect of this loss to follow-up due to euthanasia has not been assessed thoroughly. Nevertheless, as for the human scenario, the rodent’s disease profile, especially the severity of CNS infection, does not appear to represent that of the horse. As such, alternative small animal pathogenesis models should also be explored to model the equine disease.

## 8. Conclusions

Since the incursion of WNV into North America in 1999, a vast amount of investigation has taken place in characterizing the virological and immunological aspects of WNV neuroinvasion. However, several gaps in the current state of knowledge in the route and viral mechanism of WNV neuroinvasion remain (Table 7). As discussed here, clear evidence for certain hematogenous routes, such as the paracellular migration of WNV virions across the BBB, is not available. While BBB permeability appears to be associated with neuropathogenesis, its precise role is blurred by the fact that CNS infection can, in return, cause BBB disruption via the resultant cytokine release by infected neurons [53]. Appropriate delineation of the cause and effect role of the BBB and neuroinvasion is therefore an important issue to address. 

Transneural invasion by WNV also requires a closer examination, especially when there is growing evidence from *in vivo* experiments to support this [57,68,70]. However, methodological (route of inoculation), virological (strain, dose) and host (age, breed, species) variability continues to be a factor that gives rise to varying and sometimes contradicting results. The lack of consistent tissue sampling, such as the olfactory bulb and spinal cord, also obscures the overall picture of WNV neuropathogenesis, which may involve multiple routes depending on the time post-infection. This inconsistency also impedes the exploration of less well characterized barriers of the CNS, such as the BSCB. Indeed, the remaining barrier mechanisms of the brain, namely the blood-CSF and CSF-brain barriers, have not received enough attention. Recent advances in *ex vivo* methods, such as the brain slice culture, may provide new tools for investigating these avenues [143]. While the complex nature of some of these barriers, such as the arachnoid (meningeal) barrier, will present challenges for investigators to study, the shift of attention away from the popular BBB will undoubtedly lead to new insights into the neuropathogenesis of WNV.

Similarly, while the molecular determinants for the overall virulence and neuroinvasiveness of WNV strains have been studied extensively, their mechanism(s) in facilitating the process of neuroinvasion is generally poorly understood. The potential biases associated with the current methods of assessing neuroinvasiveness also need addressing. High mortality and CNS viral burden that follows peripheral viral challenge could be attributable to factors independent to the neuroinvasive potential of the virus strain. These factors include the efficiency of the virus in disseminating and productively infecting cells in the CNS. The evidence that BSCB is more permeable than BBB to potentially neurotoxic cytokines may even question the need for viral neuroinvasion for neuropathogenesis and death. Until a robust animal model with a more representative disease profile to that of humans and horses is located, translation from the popular rodent models should be performed with caution. As made evident in Table 7, the characterization of the mechanism(s) of WNV neuroinvasion is only in its infancy. With new outbreaks of WNV continuing to emerge worldwide, there is an urgency to address these gaps in knowledge in order that appropriate therapeutic and preventative strategies can be devised.

**Table 7:** Open questions in the field of WNV neuroinvasion.

Aspects of WNV neuroinvasion	Current limitations and unanswered questions
Transcytosis of virions across endothelium	Lack of *in vivo* evidence for endothelial infection by WNV
BBB permeability	Unknown whether increased permeability in the BBB precedes viral neuroinvasion or *vice versa*
Paracellular neuroinvasion by diffusion across endothelial junctions	Lack of evidence
Paracellular neuroinvasion by “Trojan Horse” method	Unknown function and trafficking behaviour of WNV infected leukocytes
Transneural neuroinvasion from peripheral somatic nerves	Use of artificial route of inoculation; Unknown whether virus can successfully reach the brain by this route;
Transneural neuroinvasion from olfactory nerves	Use of artificial intranasal inoculation
Blood-CSF barrier (choroid plexus and CVOs)	Unknown role in WNV neuroinvasion
CSF-brain barrier	Unknown role in WNV neuroinvasion
Arachnoid (meningeal) barrier	Unknown role in WNV neuroinvasion
BSCB	Unknown role in WNV neuroinvasion
Molecular determinants for the mechanism of WNV neuroinvasion	*In vivo* validation of suggested mechanisms is deficient.
Rodent models	Viremia and character of CNS infection are not representative of target hosts (human and horse). Alternative pathogenesis model should be explored.
